# Effects of eszopiclone on sleep quality and cognitive function in elderly patients with Alzheimer's disease and sleep disorder: A randomized controlled trial

**DOI:** 10.1002/brb3.2488

**Published:** 2022-01-18

**Authors:** Shuyu Huo, Lin Cheng, Shuying Li, Fulu Xu

**Affiliations:** ^1^ Department of Hospital Infection Management The Sixth Hospital of Wuhan, Affiliated Hospital of Jianghan University Wuhan China; ^2^ Outpatient Department The Sixth Hospital of Wuhan, Affiliated Hospital of Jianghan University Wuhan China; ^3^ General Department The Sixth Hospital of Wuhan, Affiliated Hospital of Jianghan University Wuhan China; ^4^ Department of Internal Medicine The Sixth Hospital of Wuhan, Affiliated Hospital of Jianghan University Wuhan China

**Keywords:** Alzheimer's disease, cognitive function, eszopiclone, sleep disorder, sleep quality

## Abstract

**Objective:**

To investigate the effects of eszopiclone on sleep quality and cognitive function in elderly patients with Alzheimer's disease (AD) and sleep disorders.

**Methods:**

This study was a prospective study of 96 elderly patients with AD and sleep disturbance treated in our hospital from April 2019 to December 2020. All patients were divided into a control group (48 patients, given alprazolam tablets) and a study group (48 patients, given eszopiclone) according to the random number table method.

**Results:**

After treatment, compared with the control group, the study group had lower sleep latency, daytime function, sleep disturbance, sleep efficiency, sleep quality, sleeping time, and hypnotic medication scores (*p* < .05). After treatment, sleep progression and sleep architecture improvement were more obvious in the study group compared with the control group (*p* < .05). After treatment, compared with the control group, the rhythm disturbance, psychotic disorder, hallucination, phobic anxiety, and disorder in the study group improved more significantly (*p* < .05). After treatment, compared with the control group, the scores of orientation, attention, memory, calculation, recall, and language ability in the study group improved more significantly (*p* < .05). After treatment, the scores of the physical life self‐care scale and instrumental activities of daily living scale in the study group were improved more obviously compared with the control group, with significant differences (*p* < .05).

**Conclusion:**

Eszopiclone can effectively improve the quality of sleep and cognitive function in elderly patients with AD and sleep disorder.

## Abstract 2

To investigate the effects of eszopiclone on sleep quality and cognitive function in elderly patients with Alzheimer's disease (AD) and sleep disorders.

Exzopiclone can effectively improve the quality of sleep and cognitive function in elderly patients with AD and sleep disorder.

## 1 INTRODUCTION

Alzheimer's disease (AD) is a common primary disorder of the central nervous system in the elderly, characterized by persistent high‐grade neurofunctional activity cognitive impairment, and is an acquired clinical syndrome of intellectual impairment, characterized by slowly progressive cognitive impairment as the main clinical feature (Breijyeh & Karaman, 2020). Patients with AD, in the absence of any disturbance of consciousness, suffer from problems with memory, mood, thinking, and spatial recognition (Zhang & Zheng, 2019).

Recently, elderly patients with AD often have sleep disorders, at the same time, which can aggravate cognitive dysfunction and social dysfunction, affect normal daily living, and reduce the quality of life (Zhou et al., 2021). Sleep disorders are currently generally recognized as an important cause of long‐term and repeated hospitalizations for treatment in elderly patients with AD (Zhou et al., 2021). Therefore, how to effectively treat AD with sleep disorder has become a key issue that needs to be addressed in the clinic.

Currently, drugs are often used in the clinic to treat AD with sleep disorders. Eszopiclone is a non‐diazepine with high safety and efficacy (Wang & Su, 2021), but its efficacy in treating AD with sleep disorder has not been reported. In this study, we investigated the effects of eszopiclone on the quality of sleep and cognitive function in elderly patients with AD and sleep disorders.

## 2 MATERIALS AND METHODS

### 2.1 Materials

This study was a prospective study. A total of 96 patients with AD and sleep disorders who were admitted to our hospital from April 2019 to December 2020 were used as the study subjects, and all patients were divided into study and control groups with 48 patients in each group according to the random number table method. The control group consisted of 20 males and 28 females, aged 62–75 years, with a mean age 67.31 ± 17.89 years, disease duration 1–9 years, mean disease duration 5.08 ± 1.19 years. Degree of dementia: mild in 17, moderate in 18, and severe in 13 patients. The study group consisted of 19 males and 29 females, aged 62–76 years, with a mean age of 67.38 ± 17.98 years, disease duration of 1–9 years, mean disease duration of 5.12 ± 1.23 years. Degree of dementia: mild in 15, moderate in 18, and severe in 15 patients. There was no difference in the comparison of general data between the two groups (*p* > .05). The protocol of this study conformed to the relevant requirements of the World Medical Association Declaration of Helsinki. This study was approved by the ethics committee of Affiliated Hospital of Jianghan University. Written informed consent was obtained from all the participating patients.

Inclusion criteria: All patients fulfilled the Diagnostic and Statistical Manual of mental disorders IV (DSM‐IV) and National Institute of Neurological and Communicative Disorders and Stroke, and the Alzheimer's disease and Related Disorders Association (NINCDS‐ADRD) established diagnostic criteria for AD (American Psychiatric Association, 1994) and fulfilled the diagnostic criteria for sleep disturbances in the International Classification of Sleep Disorders version 3 (ICSD‐3) (American Sleep Disorders Association, 1997); patients aged > 60 years.

Exclusion criteria: Had undergone other sleep intervention therapy, sleep aids, and anti‐AD pharmacological interventions within 2 weeks; had other severe multi‐organ diseases; had other psychiatric diseases; the patient's family members were blinded to the study; had poor adherence; and were allergic to the study's drugs.

## 3 METHODS

### 3.1 Therapeutic method

The patients in the control group were treated with alprazolam tablets (manufacturer: Hunan Dongting Pharmaceutical Co., Ltd.; Approval Number: H43020578) at 0.4 mg/day for 4 weeks, and the patients in the study group were treated with eszopiclone (manufacturer: Jiangsu Tianshili Diyi Pharmaceutical Co., Ltd.; Approval Number: H20070069) at 3 mg/day for 4 weeks.

### 3.2 Outcome measures

1. Sleep quality: Quality of sleep was assessed using the Pittsburgh sleep quality index (PSQI) scale before and after treatment (Zhou et al., 2006). The scale consists of seven items for sleep latency, daytime function, sleep disturbance, sleep efficiency, sleep quality, sleep duration, and hypnotic drugs and is scored on a scale of 0–3 for each index, with scores ranging from 0 to 21. Higher scores indicate worse sleep quality.
2. The degree of sleep disturbance improvement: Before and after treatment, the patients' sleep disorders were evaluated by using an eight‐channel electroencephalogram (EEG) machine (ND‐82B, China). Sleep progression and sleep architecture were recorded. Sleep architecture included sleep latency, Rapid eye movement (REM) sleep latency, total sleep time, and sleep efficiency. Sleep architecture included the sleep cycle, non‐rapid eye movement (NREM) duration, and REM sleep duration.
3. Psychiatric symptoms: Patients' psychiatric symptoms were assessed before and after treatment using the behavioral pathology in Alzheimer's disease rating scale (BEHAVE‐AD) (Wen & Yang, 2018). The scale is divided into five dimensions including rhythm disturbances, psychotic disorders, hallucinations, phobic anxiety, and disorganization, and is divided into 25 subscales, which are scored from 0 to 3. Higher scores on the scale indicate more severe psychiatric symptoms.
4. Cognitive function: The Mini‐Mental State Examination (MMSE) was used to assess patients' cognitive function before and after treatment (Gao et al., 2015). The scale consists of five items: orientation, attention, memory and calculation, recall, and language ability. Each item is answered correctly with a score of 1, incorrectly or unknowingly with a score of 0, unsuited with a score of 9, and rejected with a score of 8. When the total score is summed, both 8 and 9 points are scored 0. The maximum score is 30, with higher scores indicating better cognitive function.
5. Living ability: The activities of daily living scale (ADL) was used to assess the living ability of patients before and after treatment (Weng & Huang, 2014). The scale consists of two parts: (1) the physical life self‐care scale includes toileting, eating, dressing, grooming, walking, and bathing; (2) the instrumental ADL includes self‐care economy, laundry, housework, meal preparation, shopping, calling, using transportation, and taking medication. Each item is scored on a scale of 1–4 points, with a total score of 14–56 points, with higher scores indicating worse self‐care ability in life.

### 3.3 Statistics

The data were analyzed by SPSS 21.0 software. All the data were tested for normality distribution and homogeneity of variance before further analysis. The measurement data such as sleep quality, psychiatric symptoms, and cognitive function were met assumptions for parametric analyses and expressed in $\bar{x}\;\pm \;s$, and the statistical analysis was performed by *t*‐test. The counting data were expressed as a rate (%), and the chi‐square test was used. The *p* < .05 was considered to be statistically significant.

## 4 RESULTS

### 4.1 Eszopiclone can better improve the sleep quality of patients

We first investigated the effect of eszopiclone on the quality of patients' sleep. Before treatment, there were no significant differences in sleep latency, daytime function, sleep disturbance, sleep efficiency, sleep quality, sleeping time, or hypnotic medication scores between the two groups (*p* > .05). After treatment, the two groups' sleep latency, daytime function, sleep disturbance, sleep efficiency, sleep quality, sleeping time, and hypnotic medication scores decreased compared with those before treatment (*p* < .05). Compared with the control group, the study group had lower sleep latency, daytime function, sleep disturbance, sleep efficiency, sleep quality, sleeping time, and hypnotic medication scores (*p* < .05) (Table 1). This illustrates that both alprazolam and eszopiclone improve the quality of patients' sleep, but the effect of eszopiclone is more obvious.

**TABLE 1:** Comparison of sleep quality between the two groups (score, $\bar{x}\;\pm \;s$)

**Group**	***n***	**Stage**	**Time to fall asleep**	**Daytime function**	**Sleep disturbance**	**Sleep efficiency**	**Sleep quality**	**Sleeping time**	**Hypnotic medication scores**
Control	48	Before	1.83 ± 0.19	1.81 ± 0.17	1.82 ± 0.16	1.82 ± 0.18	1.82 ± 0.17	1.83 ± 0.18	1.83 ± 0.19
		After	0.67 ± 0.01*	0.76 ± 0.02*	0.79 ± 0.03*	0.77 ± 0.02*	0.73 ± 0.03*	0.78 ± 0.02*	0.76 ± 0.03*
Study	48	Before	1.87 ± 0.21	1.82 ± 0.19	1.89 ± 0.23	1.87 ± 0.21	1.84 ± 0.19	1.85 ± 0.17	1.86 ± 0.18
		After	0.21 ± 0.02* *^,^* *^#^*	0.23 ± 0.03* *^,^* *^#^*	0.25 ± 0.02* *^,^* *^#^*	0.22 ± 0.03* *^,^* *^#^*	0.21 ± 0.02* *^,^* *^#^*	0.19 ± 0.02* *^,^* *^#^*	0.18 ± 0.02* *^,^* *^#^*
Comparison between groups after treatment		*t* value	−142.526	−101.842	−103.763	−105.685	−99.920	−144.52	−111.449
		*p*	< .001	< .001	< .001	< .001	< .001	< .001	< .001

* Compared with before treatment, *p* < .05.

^#^ Compared with control group, *p* < .05.

### 4.2 Eszopiclone resulted in more significant alleviation of the patient's sleep disturbance

We also studied the condition of sleep disturbance in our patients. Before treatment, there were no significant differences in sleep progression or sleep architecture between the two groups (*p* > .05). After treatment, sleep progression and sleep architecture in the two groups improved compared with those before treatment (*p* < .05). And sleep progression and sleep architecture improvement were more obvious in the study group compared with the control group (*p* < .05) (Table 2). This illustrates that both alprazolam and eszopiclone can relieve the patient's sleep disturbance, but the eszopiclone effect is more obvious.

**TABLE 2:** Comparison of remission of sleep disorder between two groups ( $\bar{x}\;\pm \;s$)

			**Sleep progression**				**Sleep architecture**		
**Group**	***n***	**Stage**	**Sleep latency (min)**	**REM latency (min)**	**Total sleep time (min)**	**Sleep efficiency (%)**	**Sleep cycle (min)**	**NREM time (min)**	**REM sleep time (min)**
Control	48	Before	48.79 ± 9.12	105.28 ± 12.03	290.81 ± 22.09	58.93 ± 12.31	6.94 ± 0.32	214.29 ± 13.63	52.03 ± 12.39
		After	25.38 ± 6.42*	113.09 ± 12.32*	408.93 ± 33.29*	80.93 ± 7.49*	4.98 ± 0.21*	301.98 ± 18.29*	69.09 ± 12.01*
Study	48	Before	48.83 ± 9.07	92.13 ± 11.39	289.98 ± 21.29	58.81 ± 11.91	6.98 ± 0.34	212.98 ± 13.29	51.29 ± 11.39
		After	21.09 ± 6.38* *^,^* *^#^*	126.82 ± 12.09* *^,^* *^#^*	418.98 ± 34.98* *^,^* *^#^*	87.93 ± 7.39* *^,^* *^#^*	4.12 ± 0.19* *^,^* *^#^*	339.87 ± 15.39* *^,^* *^#^*	78.92 ± 12.09* *^,^* *^#^*
Comparison between groups after treatment		*t* value	−3.284	5.511	2.703	4.609	−20.742	10.982	3.996
		*p*	.001	< .001	.008	< .001	< .001	< .001	< .001

*Abbreviations*: NREM, non‐rapid eye movement; REM, rapid eye movement.

* Compared with before treatment, *p* < .05.

^#^ Compared with control group, *p* < .05.

### 4.3 After eszopiclone treatment, the patient's mental symptoms were significantly relieved

Psychiatric symptoms such as fear and anxiety are frequent in AD patients with sleep disorders, so we assessed the remission of psychiatric symptoms in our patients. Before treatment, there were no differences in rhythm disturbances, psychotic disorders, hallucinations, phobic anxiety, or disorders between the two groups (*p* > .05). After treatment, the rhythm disturbance, psychotic disorder, hallucination, phobic anxiety, and disorder in the two groups were improved compared with those before treatment (*p* < .05). Compared with the control group, the rhythm disturbance, psychotic disorder, hallucination, phobic anxiety, and disorder in the study group improved more significantly (*p* < .05) (Table 3). This confirms the remarkable efficacy of eszopiclone in alleviating psychiatric symptoms in such patients.

**TABLE 3:** Comparison of psychiatric symptoms between the two groups (score, $\bar{x}\;\pm \;s$)

**Group**	***n***	**Stage**	**Rhythm disturbances**	**Psychotic disorder**	**Hallucinations**	**Phobic anxiety**	**Disorder**
Control	48	Before	9.81 ± 0.36	9.87 ± 0.38	9.87 ± 0.37	9.79 ± 0.23	9.89 ± 0.32
		After	1.98 ± 0.15*	1.91 ± 0.13*	1.89 ± 0.11*	1.90 ± 0.12*	1.93 ± 0.11*
Study	48	Before	9.87 ± 0.38	9.98 ± 0.35	9.89 ± 0.36	9.85 ± 0.32	9.92 ± 0.35
		After	1.37 ± 0.13*, *^#^*	1.32 ± 0.14*, *^#^*	1.38 ± 0.12*, *^#^*	1.42 ± 0.15*, *^#^*	1.32 ± 0.11*, *^#^*
Comparison between groups after treatment		*t* value	−21.291	−21.396	−21.705	−17.312	−27.167
		*p*	< .001	< .001	< .001	< .001	< .001

* Compared with before treatment, *p* < .05.

^#^ Compared with control group, *p* < .05.

### 4.4 Eszopiclone can significantly improve the cognitive function of patients

In terms of cognitive function, before treatment, there were no differences in the scores of orientation, attention, memory and calculation, recall, and language ability between the two groups (*p* > .05). After treatment, the scores of orientation, attention, memory, calculation, recall, and language ability of the two groups were improved compared with those before treatment (*p* < .05). Compared with the control group, the scores of orientation, attention, memory, calculation, recall, and language ability in the study group improved more significantly (*p* < .05) (Table 4). This proves that eszopiclone has a better effect on improving cognitive function in such patients than alprazolam.

**TABLE 4:** Comparison of cognitive function between two groups (score, $\bar{x}\;\pm \;s$)

**Group**	***n***	**Stage**	**Orientation**	**Attention**	**Memory and calculation**	**Recall**	**Language ability**
Control	48	Before	4.00 ± 0.19	3.98 ± 0.17	4.06 ± 0.13	4.01 ± 0.13	4.01 ± 0.12
		After	1.23 ± 0.09*	1.21 ± 0.11*	1.19 ± 0.14*	1.13 ± 0.13*	1.15 ± 0.12*
Study	48	Before	4.01 ± 0.21	4.03 ± 0.19	4.09 ± 0.17	4.03 ± 0.16	4.03 ± 0.13
		After	0.63 ± 0.12*, *^#^*	0.61 ± 0.13*, *^#^*	0.62 ± 0.09*, *^#^*	0.69 ± 0.07*, *^#^*	0.63 ± 0.04*, *^#^*
Comparison between groups after treatment		*t* value	−27.713	−24.410	−23.728	−20.646	−28.482
		*p*	< .001	< .001	< .001	< .001	< .001

* Compared with before treatment, *p* < .05.

^#^ Compared with control group, *p* < .05.

### 4.5 The patient's living ability significantly improved after eszopiclone treatment

The self‐care ability of such patients in life directly affects their quality of life, so we also evaluated the effect of eszopiclone on their viability. Before treatment, there were no differences in the scores of the physical life self‐care scale and instrumental ADL between the two groups (*p* > .05). After treatment, the scores of the physical life self‐care scale and instrumental ADL in the two groups were improved, compared with those before treatment (*p* < .05), and the scores of the physical life self‐care scale and instrumental ADL in the study group were improved more obviously compared with the control group, with significant differences (*p* < .05) (Table 5).

**TABLE 5:** Comparison of living ability between two groups (score, $\bar{x}\;\pm \;s$)

**Group**	***n***	**Stage**	**Physical Life self‐care scale**	**Instrumental activities of daily living scale**
Control	48	Before	18.79 ± 1.19	20.19 ± 1.39
		After	9.21 ± 1.03*	11.04 ± 1.09*
Study	48	Before	18.87 ± 1.21	21.09 ± 1.43
		After	7.09 ± 1.02*, #	9.07 ± 1.03*#
Comparison between groups after treatment		*t* value	−10.132	−9.101
		*p*	< .001	< .001

* Compared with before treatment, *p* < .05.

^#^ Compared with control group, *p* < .05.

### 4.6 The incidence of adverse reactions in the two groups

The incidence of adverse reactions in the observation group was lower than that in the control group (*p* < .05) (Table 6).

**TABLE 6:** The incidence of adverse reactions in the two groups (*n*, %)

**Group**	***N***	**Fatigue**	**Bitter**	**Dizziness**	**Drunk**	**Vomit**	**Total adverse reaction rate**
Control	48	4	6	4	1	1	16 (33.33)
Study	48	2	2	1	1	1	7 (14.58)
χ^2^							4.631
*p*							.031

## 5 DISCUSSION

AD can be caused by various factors, such as cardiovascular disease, endocrine disorders, genetics, and viral infections, and as the integration of brain upper and lower motor neurons with extrapyramidal and cerebellar systems becomes dysfunctional, patients can experience cognitive dysfunction, which can affect sleep quality (Naseri et al., 2019). Poor sleep quality has a large adverse effect on the improvement of AD prognostic status, and behavioral abnormalities in aspects such as emotional cognition due to AD can worsen sleep quality and induce sleep disorders. About 44% of AD patients have sleep disorders. Moreover, sleep disorders can aggravate cognitive dysfunction and behavioral abilities, aggravate psychiatric symptoms, and accelerate the course of brain functional decline in AD patients (Villain & Dubois, 2019). Patients with AD have a variety of dysfunctions such as memory, language, and cognition. Therefore, it is clinically important to intensify the aggressive treatment of sleep disorders while treating AD, to be able to improve its clinical symptoms and the level of prognosis.

In this study, both groups of patients were routinely treated with donepezil hydrochloride, which is a second‐generation cholinesterase inhibitor that can selectively inhibit the hydrolysis of acetylcholine in the central nervous system, increase the concentration of acetylcholine, and improve the function of the brain choline system and cognitive function of patients, and is a long‐acting symptomatic treatment drug for AD (Dubois et al., 2012; Zhang et al., 2018). Currently, sedative hypnotic drugs, such as clonazepam and estazolam, are often used in the clinic to treat sleep disorders in AD patients, and this class of drugs has anti‐anxiety and depression effects, which can effectively improve the quality of sleep in patients, but such drugs may cause over sedation, affect the cognitive function of patients, and easily produce drug dependency, etc., which is not conducive to clinical promotion (Zhang et al., 2021). Alprazolam is a benzodiazepine drug, the mechanism of which is to bind to the benzodiazepine receptor coupled gamma‐aminobutyric acid (GABA) complex, which helps the patients to calm down through a mechanism that affects the function of the limbic system, that produces a hypnotic effect (Du et al., 2019). However, studies have pointed out that alprazolam may have a hangover effect and have certain effects on cognition, memory, and motor functions. Therefore, new and independent non‐benzodiazepine drugs for improving sleep have attracted more and more clinical attention. Eszopiclone belongs to a non‐benzodiazepine class of drugs. It is a commonly used clinical medicine for the treatment of sleep disorders, and it has achieved good results in patients with mental illness sleep disorders. (Huang, 2021; Wang et al., 2015). Eszopiclone was approved by the United States Food and Drug Administration (US FDA) in December 2014 for the treatment of sleep disorders with fewer adverse effects. It is able to effectively shorten the sleep latency of patients, prolong the sleep time and total sleep time of the N3 stage, can effectively improve the quality of patients' sleep, reduce the number of nocturnal awakenings, and have less effect on patients' daytime function. This was the reason why eszopiclone was chosen for this study to treat elderly patients with dementia and sleep disorders. Among various drugs to treat sleep disorders, eszopiclone has more advantages. It has a rapid onset and duration of action of 5 to 6 h, and the patients' hangover manifestations and drug residual effects after drug administration are all milder, with significantly fewer nocturnal awakenings, no obvious drug resistance after long‐term administration, and fewer toxic effects (Trauer et al., 2015). In addition, eszopiclone is a new type of anti‐sleep disorder drug, which belongs to the class of cyclopyrrolones. Previous studies have confirmed that eszopiclone has a shorter time to peak than zopiclone, and its active ingredient half‐life is shorter (Xu et al., 2019). The hypnotic sedative effects of eszopiclone on the central nervous set the stage for the treatment of sleep disorders, and its pharmaceutical advantages are closely related to the regulation of sleep‐related neurotransmitter expression by this drug.

The PSQI scale was proposed by Buysse et al. in 1989, which has become a commonly used scale for clinical assessment in psychiatric departments (Bao, 2017). The results of this study showed that, compared with the control group, the study group had lower scores for time to fall asleep, daytime function, sleep disturbance, sleep efficiency, sleep quality, sleeping duration, and hypnotic drugs, suggesting that eszopiclone can effectively improve the quality of sleep in patients with AD and sleep disorders. This may be due to the ability of eszopiclone to effectively repair neurotransmitters, which has a positive effect on the dysregulated expression of neurotransmitters resulting from neurodegeneration in such patients. Zhu et al.’s study confirmed that eszopiclone was more effective than estazolam in the treatment of hypertension with sleep disturbance, and it had fewer adverse effects such as drowsiness, daytime sleepiness, and hangover (Zhu et al., 2020), which was similar to the results of this study. The research results of Wang et al. (2017) showed that eszopiclone can effectively shorten the time to fall asleep, increase the total sleep time, and improve the quality of sleep, similar to the results of this study.

Polysomnography enables the simultaneous recording and analysis of multiple sleep physiological indexes for research in sleep medicine and basic sleep diagnosis (Wang et al., 2007). The comprehensive polysomnography includes many kinds of physiological detectors, including EEG, electromyogram (EMG), oculogram, plethysmograph, and electrocardiogram (ECG), is able to comprehensively collect many kinds of physiological changes in sleep, can effectively evaluate the degree of sleep disturbance, and its quantitative analysis is of great significance to evaluate the effectiveness of sleep. The results of this study showed that after treatment, the sleep progression and sleep structure of patients in the two groups were improved compared with those before treatment, and the improvement of sleep progression and sleep structure was more obvious in the study group compared with the control group, suggesting that eszopiclone can effectively alleviate sleep disorders in such patients. Huang et al. found that eszopiclone was safe and effective in improving sleep parameters in elderly patients with AD and sleep disorder (Trauer et al., 2015), which is consistent with the findings of this study. In this study, we further analyzed the pathological behaviors of elderly patients with AD and sleep disorder, and the results showed that the improvement of rhythm disturbances, psychotic disorders, hallucinations, phobic anxiety, and disorders was more obvious in the study group compared with the control group, suggesting that eszopiclone could effectively relieve the pathological behaviors of patients with AD and sleep disorder. This may be due to the ability of eszopiclone to improve the quality of sleep in these patients, and the specific mechanism needs to be confirmed by further research.

The MMSE has been widely used to screen and aid in the diagnosis of AD by using convenient and multiple measurement items, simple scoring, and high sensitivity (Jian et al., 2014). Research by Zou, 2019 and others pointed out that eszopiclone and alprazolam are equivalent in improving AD with sleep disorders, but the incidence of adverse reactions of eszopiclone is lower, and it also helps to improve patients’ cognitive function and activity ability. The results of this study showed that during the treatment process, the two groups of patients had adverse reactions such as fatigue, bitter mouth, and dizziness. The incidence of adverse reactions in the observation group was lower than that in the control group (*p* < .05). The degree of adverse reactions in both groups was mild, and the symptoms disappeared spontaneously without any intervention. The results of this study showed that, after treatment, scores for orientation, attention, memory and calculation, recall, and language were improved more significantly in the study group compared with the control group, indicating that eszopiclone is effective in improving cognitive function in patients with AD and sleep disorders. Moreover, eszopiclone was also effective in improving the living ability of these patients.

### 5.1 Limitations

This study has several limitations. First, the sample size of the control group and the study group is relatively small. Studies with a larger sample size should be conducted to further confirm the conclusions in this study. Second, the data in this study is collected from a single center, which may be only representative of patients in the central region of China. More studies with multiple centers should be performed in the future. Third, the mechanisms of eszopiclone on sleep quality and cognitive function has not been clarified in this study, which is the next research plan of our group.

## 6 CONCLUSION

In summary, it is suggested that dexzopiclone can effectively improve the sleep quality of patients with Alzheimer's and sleep disorders, help improve the degree of sleep disorders, and restore the patients' mental symptoms and cognitive functions, improve living ability, and have higher safety. It is worthy of clinical application.

## CONFLICT OF INTEREST

The authors declare no conflict of interest.

### PEER REVIEW

The peer review history for this article is available at https://publons.com/publon/10.1002/brb3.2488

## Data Availability

The datasets generated and analyzed during the current study are available from the corresponding author on reasonable request.
